# Implementation and Application of PSF-Based EPI Distortion Correction to High Field Animal Imaging

**DOI:** 10.1155/2009/946271

**Published:** 2009-12-31

**Authors:** Dominik Paul, Maxim Zaitsev, Laura Harsan, Anja Kurutsch, Daniel Nico Splitthoff, Franciszek Hennel, Morwan Choli, Dominik von Elverfeldt

**Affiliations:** ^1^Department of Diagnostic Radiology, Medical Physics, University Hospital Freiburg, 79106 Freiburg, Germany; ^2^Institute of Pharmaceutical Sciences, Albert-Ludwigs-University Freiburg, 79104 Freiburg, Germany; ^3^Bruker Biospin, Ettlingen, Germany; ^4^Research Center Magnetic Resonance Bavaria e.V (MRB), 97074 Würzburg, Germany

## Abstract

The purpose of this work is to demonstrate the functionality and performance of a PSF-based geometric distortion correction for high-field functional animal EPI. The EPI method was extended to measure the PSF and a postprocessing chain was implemented in Matlab for offline distortion correction. The correction procedure was applied to phantom and in vivo imaging of mice and rats at 9.4T using different SE-EPI and DWI-EPI protocols. Results show the significant improvement in image quality for single- and multishot EPI. Using a reduced FOV in the PSF encoding direction clearly reduced the acquisition time for PSF data by an acceleration factor of 2 or 4, without affecting the correction quality.

## 1. Introduction

Due to its short acquisition time and high temporal resolution, echo-planar imaging (EPI) is often the method of choice for data acquisition in fast imaging including diffusion-weighted imaging (DWI), diffusion tensor imaging (DTI), perfusion MRI, and functional MRI (fMRI). 

However, EPI is extremely sensitive to magnetic field inhomogeneities (e.g., close to tissue/bone or tissue/air interfaces) because of the prolonged readout and hence low bandwidth in the phase encoding (PE) direction [1]. This results typically in geometric and image intensity distortions. In addition to time-invariant off-resonance effects such as B0 field inhomogeneities, dynamic field variations induced by eddy currents may further contribute to the observed image distortions. 

Several methods to overcome limitations of geometric image distortions can be found in literature [2, 3]. Techniques based on the field mapping approach—though elegant and intuitive—face implementation difficulties related to the need for phase unwrapping, particularly at high fields and when imaging small objects. Multireference methods [4, 5] offer a greater degree of stability at expense of prolonged imaging time. Among these methods, the Point Spread Function- (PSF-) based approach for distortion correction (DiCo) by Robson et al. is often used in MRI [6]. Using an extended EPI acquisition with an additional gradient encoding on the phase encoding (PE) axis, the PSF can be measured and calculated for each point in image space. Based on the PSF information, a pixel shift map (PSM) can be calculated and applied to conventional EPI data for image distortion correction. 

The PSF mapping is a very robust method and allows the quantification of distortions in regions of low- and high-field inhomogeneities. In addition, EPI inherent eddy currents and concomitant gradients cause identical distortions of the PSF data as in EPI and, thus, are also mapped faithfully [7]. 

The acquisition steps for the PSF data depends directly on the matrix size and can result in a substantial acquisition time increase. However, there are several further developments to speed up PSF mapping including the application of parallel imaging techniques (e.g., GRAPPA [8]) and the reduced Field of View (rFOV) approach to PSF data acquisition [7]. With regard to postprocessing, the calculation of the pixel shift map is computationally cheap and fast and shows a high stability. Another reason for the choice of the PSF-based method is several years of the positive experience with the method in routine human EPI examinations at our 3T systems. 

In this paper we present an implementation of the PSF-based distortion correction methodology on an animal imaging system and its application to EPI at high fields. The inaccuracies of the phase information in EPI—and hence, the geometric distortions—increase with the field strength and if measured in pixels, the geometric distortions increase in an over-proportional manner at higher resolutions that are typical for high-field animal MRI. Thus, the aim of this work is to investigate the performance of the PSF-based correction method for high-field EPI applications in animal MRI. Unfortunately EPI distortion correction in animal MR imaging has received extremely scant attention of the research community [9], and the fair comparison between different known correction methods given the special requirements of animal imaging has not been performed elsewhere. Therefore the choice was made to implement the PSF technique, which has proven its stability over several years of experience at our institution [7] and was also recommended by previous publications from other groups [1].

## 2. Methods

### 2.1. PSF Data Acquisition

**Figure 1:**
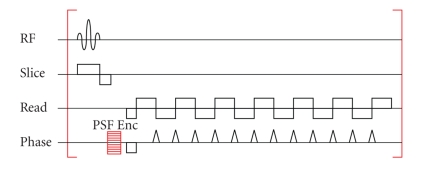
EPI pulse sequence with additional PSF encoding gradient (PSF Enc) on the phase encoding axis. In order to acquire the complete PSF data, EPI acquisitions are repeated for the complete PSF Encoding gradient table.

For the acquisition of PSF data, a standard EPI method was extended by an additional gradient encoding on the phase encoding axis as described in [6]. The resulting pulse sequence diagram is given in Figure 1.

In addition, the method was adapted such that raw data (later called “PSF raw data”) are stored after all postprocessing steps (e.g., ramp sampling correction, GRAPPA reconstruction of missing k-space lines, etc.) but before image reconstruction. The raw data were stored for all receiver channels separately. The described method was implemented on a Bruker Biospin 94/20 system in ParaVision 5 (Bruker BioSpin, Ettlingen, Germany). 

In addition, our implementation allowed for automatic PSF encoding settings (gradient strength and number of repetitions) and acquisition time reduction using rFOV.

### 2.2. PSF Data Postprocessing

**Figure 2:**
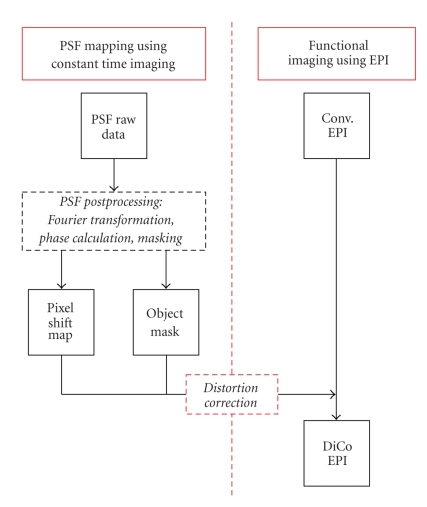
PSF data acquisition and postprocessing chain for EPI distortion correction. PSF data are acquired and a Pixel Shift Map (PSM) calculated. The PSM is later applied on conventional EPI inside the image mask in order to improve image quality (right side of flow diagram).

Postprocessing of PSF raw data was performed offline on a standard PC running Fedora Linux (Fedora 8) using the Matlab software package (Matlab R2007b, the Mathworks Inc., Natick, MA, USA). The different steps for data postprocessing are illustrated in Figure 2.

The first step includes reading the PSF raw data. The PSF raw data can be considered as a 3D data set with dimensions of EPI frequency, EPI phase encoding, and PSF encoding. 

In order to get complex image data a two-dimensional Fourier transformation is applied in EPI frequency and phase encoding directions. In the case of multichannel data, this step is performed separately for each coil element and the resulting images are combined complex using *adaptive reconstruction *from Walsh et al. [10] in order to preserve the phase information necessary for the following processing steps. 

In order to calculate the PSF, a Fourier transformation is applied in the third dimension (PSF encoding direction). Next, the shift of the PSF is determined to subpixel resolution using the Fourier shift theorem [7, 11] for every point in image space. This results in a pixel shift map (PSM), which is later used to correct for image distortions. 

Integrating the PSF image intensity along the distorted dimension yields a nondistorted image [7]. The nondistorted image has the same echo time and, thus, image contrast as the corresponding echo-planar image. It represents a spin echo (SE) or gradient echo (GE) image corresponding to whether an SE-EPI or GE-EPI is used. In addition, an object mask is generated from the nondistorted image using threshold segmentation that is later used during the distortion correction procedure.

### 2.3. Distortion Correction

In the last postprocessing step, conventional EPI data (e.g., fMRI or DWI) are corrected for geometric image distortions. Therefore, a pixel inside the object mask retrieves a value determined by its original position and the corresponding shift in the pixel shift map from the original image (EPI). Due to the subpixel shifts present in the pixel shift map, a piecewise cubic spline interpolation procedure was used to determine the corrected pixel intensity values. 

As stated above, this operation is only performed for pixels inside the object mask to prevent artifact generation outside of the distortion-corrected object. Note that the data inside of the object mask cannot be damaged by incorrect mask generation. 

The corrected image data are stored inside the original data folder as an additional reconstruction for this examination.

### 2.4. Experiments

The distortion correction was applied to conventional spin-echo (SE-EPI) and gradient-echo (GE-EPI) echo-planar imaging on phantoms as well as to functional magnetic resonance imaging with diffusion-weighted echo-planar imaging (DW-EPI) on mice and rats in vivo. All experiments were performed on a 9.4T Bruker Biospin animal system equipped with a BGA12S gradient system capable of 675 mT/m. A transmit/receive 1H mouse quadrature birdcage resonator with an inner diameter of 35 mm was used for mice and phantom imaging. For rat imaging, a transmit 1H quadrature birdcage resonator with an inner diameter of 70 mm was used for RF excitation and a 4-channel phased array head coil was used for signal detection. 

For phantom experiments, a phantom was built using a plastic cylinder with an inner diameter of 30 mm (Falcon Tube). The tube was filled with two different agarose concentrations (inner part: 1%; outer part 2%) in order to provide contrast between the two compartments. 

Acquisition parameters for a single-shot SE-EPI were TR = 3000 milliseconds, TE = 52.18 milliseconds, BW = 200 kHz, and 10 repetitions. Ten consecutive slices with 1 mm slice thickness, an FOV of 40 mm × 40 mm, and a matrix size of 192 × 96 were acquired in 30 seconds. Total acquisition time for PSF data with 96 images for each slice was 4 : 48 minutes. 

For in vivo data acquisition, animals were narcotized using an isofluran-oxygen mixture (~2Vol% isofluran for rats, ~2.5Vol% isofluran for mice). ECG, respiration, and core temperature of the animals were monitored during the experiments. An external heating bath was used to prevent body cooling. All experiments were in concordance with the local ethics committee (approval G-08/54). 

Acquisition of mice images was performed using a single-shot, diffusion-weighted SE-EPI, and the following parameters: TR = 4000 milliseconds, TE = 18.3 milliseconds, and BW = 400 kHz. Geometric settings were 8 consecutive slices, 0.6 mm slice thickness, FOV = 30 mm × 20 mm, and 96 × 64 image matrix, resulting in an isotropic in-plane resolution of 0.3125 × 0.3125 mm. However, in order to reduce SE-EPI acquisition duration, partial Fourier encoding with an acceleration factor of 1.6 and 8 overscan lines was employed. Hence, the acquisition matrix was reduced to 96 × 40. Five b = 0 s/mm² images without diffusion weighting and 34 diffusion-weighted images were acquired in TA = 2 : 36 minutes. For diffusion weighting, different b-values ranging from 100 to 3400 s/mm² were applied.

Acquisition parameters for PSF data using conventional SE-EPI were TR = 3000 milliseconds and TE = 8.8 milliseconds, with the geometric settings equal to the respective parameters in DW-EPI. For each slice, 64 images were acquired in TA = 3 : 12 minutes. In this context, the reduction of TR also reduced the TA. 

Diffusion-weighted rat images were acquired with a 4-shot SE-EPI, and parameters of TR = 3000 milliseconds, TE = 17 milliseconds, and BW = 300 kHz. Geometric settings were 12 slices, 0.5 mm slice thickness, FOV = 40 mm × 40 mm, and 128 × 96 matrix size. Partial Fourier with an acceleration factor of 1.2 and 21 overscan lines was used. In addition to one b = 0 image, 5 diffusion-weighted images were acquired with b = 200, 400, 600, 800, and 1000 s/mm². 

In order to derive correct distortion information, PSF data were also acquired using a 4-shot SE-EPI sequence. For full PSF data sampling, this resulted in an acquisition time of 19 : 12 minutes. 

To shorten acquisition time for PSF data acquisition, the rFOV functionality was utilized, as described in [3]. Two additional PSF datasets were acquired with an rFOV acceleration factor of 2 and 4. This reduced the PSF data acquisition time from 19:12 minutes to 9:36 minutes (acceleration factor of 2) and 4 : 48 minutes (acceleration factor of 4), respectively. The resulting pixel shift maps and distortion corrected images using the PSF data with rFOV were compared to results calculated from full PSF data. 

All EPI data were transferred to the offline Linux-PC for distortion correction and stored on the acquisition computer for further postprocessing. This included the calculation of ADC maps directly in ParaVision for the original and corrected datasets.

## 3. Results

**Figure 3:**
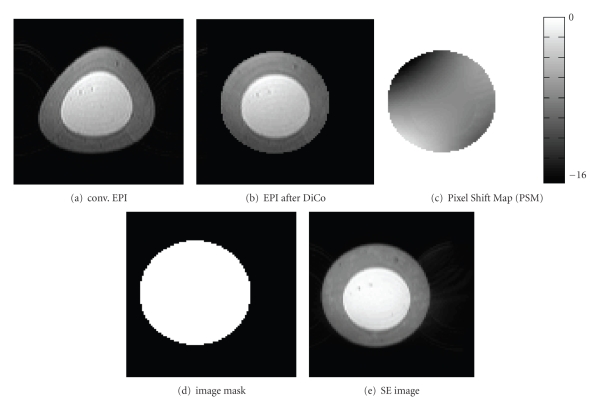
Results from the application of the distortion correction on a phantom. (a) Conventional SE-EPI. The cylindrical object is clearly deformed. (b) SE-EPI after DiCo shows improved geometric properties of the phantom. (c) Pixel Shift Map calculated from PSF data and applied to the EPI during the correction process. In addition, the object mask (d) and a nondistorted Spin Echo (e) image are shown, which were calculated from the PSF data. Note that ghosting artifacts are visible outside the phantom in conventional EPI (a). Due to the application of the correction process inside the object mask (d) no ghosting can be identified in (b).

Figure 3 shows exemplary results for distortion correction on phantoms using SE-EPI. The upper row shows the conventional SE-EPI (a) in comparison to the distortion corrected SE-EPI (b). The computation time for distortion correction was below 1 minute. The nondistorted SE image (c) and corresponding image mask (d) are given in the lower row. These images were directly obtained from the PSF data. In addition, Figure 3(e) shows the Pixel Shift Map (PSM) with shifts from black (−16 Pixels) to white (0 Pixels). The shim values and B0 settings were adjusted to increase the effect on geometric distortions. 

**Figure 4:**
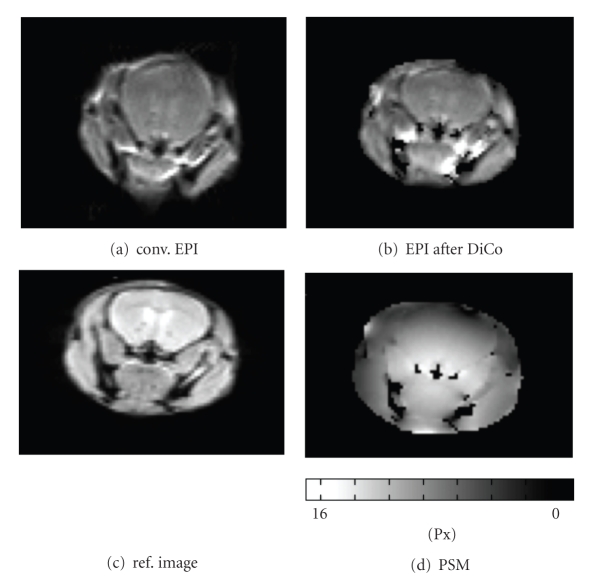
Exemplary results from diffusion-weighted EPI (DWI-EPI with b = 600 s/mm^2^) acquired in mice (in vivo). The conventional EPI (a) shows strong distortions and an “inflated” brain. The corresponding corrected image (b) shows a clearly improved representation of the brain area. The nondistorted image (c) and the PSM (d) are given in the lower row. Note that an artifact in the distortion corrected image in the area without PSM information. However, the artifact is outside the brain area and does not influence other structures.

The correction of in vivo DW-EPI mice data is presented in Figure 4. The conventional DW-EPI image (a) shows strong distortions in the head resulting in an “inflated” brain. In contrast, the distortion-corrected image (b) looks similar to the nondistorted SE image (c), which was directly calculated from the PSF data and used to generate the image mask. Figure 4(d) shows the corresponding PSM with shift values ranging from 16 (white, only inside image mask) to 0 (black). 

**Figure 5:**
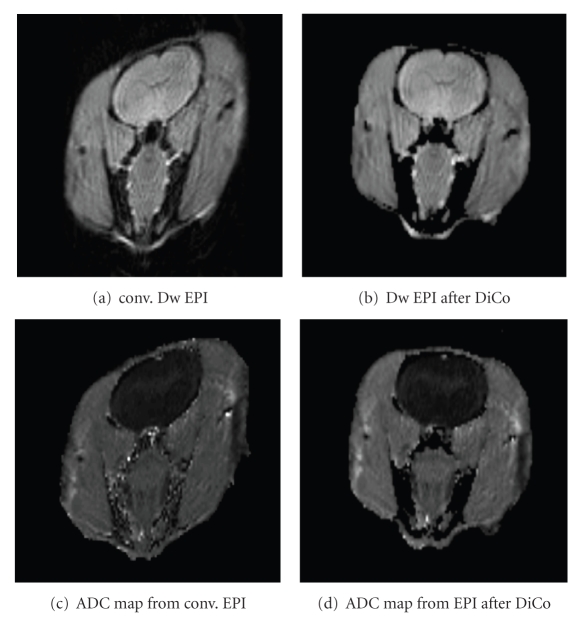
Example of diffusion weighted image acquisition in a rat. A 4-shot diffusion weighted SE-EPI was used for data acquisition. (a) Original SE-EPI and (b) SE-EPI after DiCo. ADC maps were calculated from original and corrected SE-EPI and are given in (c) and (d). In the SE-EPI (b) and corresponding ADC map (d) a significant improvement in geometric representation can be seen.

Figure 5 shows exemplary results of the distortion correction applied on multishot DW-EPI in rats. The conventional DWI-EPI (Figure 5(a)) shows strong image distortions, which were corrected using the described method (Figure 5(b)). The resulting ADC maps are given in Figure 5(c) calculated from the original DW-EPI and in Figure 5(d) calculated from the corrected DW-EPI. 

**Figure 6:**
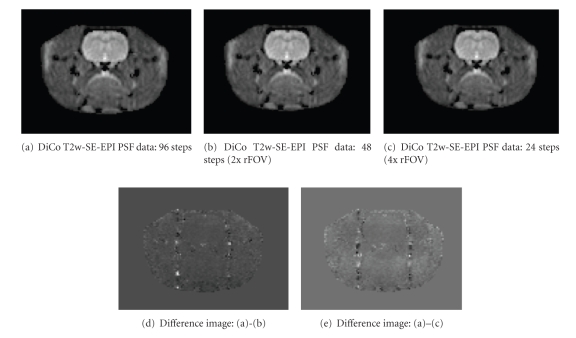
Comparison of results from EPI distortion correction using accelerated PSF data acquisition with reduced FOV. Image (a) shows a T2w SE-EPI after distortion correction using a PSM from full PSF data acquisition. For comparison, (b) and (c) show results from the correction process using PSF data with 2x and 4x acceleration. No difference in image quality can be seen. The difference images between images (a)-(b) and (a)–(c) are given in the lower row as (d) and (e), respectively.

Results from the application of the rFOV option are given in Figure 6. The upper row shows distortion-corrected T2w SE-EPI using different PSMs. The PSMs were calculated from full PSF data acquisition (Figure 6(a)) and with an acceleration factor of 2 (Figure 6(b)) and 4 (Figure 6(c)) using the rFOV functionality. No difference can be seen in image quality. In addition, the lower row shows the difference between Figure 6(a) and the images with acceleration (Figures 6(b) and 6(c)). Major differences can only be seen outside the brain.

## 4. Discussion

This paper presents our work on the implementation and application of a PSF-based distortion correction for echo-planar imaging as used in functional animal imaging. The method works reliably and delivers a significant increase in image quality while correcting for geometric image distortions. Even readjusting the shim settings did not affect the performance and stability of the correction procedure. 

While the PSF data acquisition is directly integrated into the EPI method, the DiCo postprocessing is performed offline on an additional Linux-computer. Further and more intensive usage of this method may motivate a direct implementation in the ParaVision reconstruction pipeline. In this case the correction procedure can be performed on the acquisition computer automatically during the reconstruction process and the corrected and uncorrected datasets can both be stored as independent reconstructions. The additional postprocessing time would be on the order of seconds. In this case, copying the data to the offline PC, manual start of the correction and transferring the data back to the acquisition computer would not be necessary. This would also allow the use of the correction method routinely without much effort in order to increase the workflow performance. In this context, we would like to mention our quality experience in using the method on our human 3T system where the PSF distortion correction is applied completely automatic for all routine EPI acquisitions. 

It is appropriate to compare the PSF mapping technique to the most commonly used distortion correction approach based on field mapping [2, 3]. The field mapping technique is built upon a solid theoretical basis (3) and is in principle capable of correcting local pixel shifts in both the read-out and phase-encode directions. However, due to the high bandwidth in the read-out direction and alternating readout polarities, distortions are mainly affecting the phase-encoding direction, where the PSF-based methods provide accurate corrections [1, 7, 12]. Although field mapping is a relatively fast and easy to implement technique, the need to balance between field map SNR and resolution and the phase unwrapping complexity has prevented it from reaching the robustness required for day-to-day practice. Specifically, at the high field of 9.4T and in small animals, phase unwrapping may cause significant difficulties. In addition, field mapping ignores the time-dependent magnetic field variations such as those caused by eddy currents and replaces the true field evolution with a value estimated at the echo time. Since the PSF method is based on the original EPI readout timing, the inherent eddy currents and concomitant gradients cause identical distortions of the PSF data as in EPI and, thus, are also mapped faithfully [7]. 

Animal movement during data acquisition or between the acquisition of PSF data and EPI data presents a limitation for the current implementation. However, this is a common problem of all pre- or postscan-based correction methods where the information of distortions is sampled before or after the functional imaging. In order to overcome this limitation one may use a proper fixation of the animals or image-based motion correction. 

Depending on geometric parameters, the acquisition time of the PSF reference scan may be long. However, in comparison to the prolonged acquisition times of DTI experiments, the additional time on the order of a couple of minutes is not significant and can surely be accepted. In this context, it is also to be noted that shimming may sometimes take several minutes. In fMRI, there is often a time gap during which the animal is lead over from isofloran anesthesia to paralysis using meditomedin [13]. This time gap is on the order of 20 to 40 minutes, and during this gap no fMRI acquisition can be performed. However, this time gap is more than enough for the acquisition of the PSF data, even without using the rFOV functionality. If the acquisition time for PSF data is still too long, there are several methods to further reduce it. Parallel imaging can be applied also in the PSF encoding direction and depending on the coil settings and other RF channels; an acceleration of 2 and more can be applied. In addition, this time saving method can be combined with the rFOV functionality without any limitations [7]. Other possibilities for the acceleration include the reduction of TR. While the contrast of the resulting images is not the determining factor for the calculation of the PSM, a T1-weighted PSF data acquisition is sufficient for the distortion correction of T2w-, DWI-, or fMRI-EPI as long as bandwidth and geometry settings are not affected. 

In this study, the acceleration of PSF data acquisition using rFOV showed no major differences in later distortion corrected EPI. Smaller differences were identified, but all were located outside the brain and on parallel lines in the PE direction on both sides of the throat. These differences were not discovered in any phantom experiments. We assume that these differences are derived from different PSF data due to the breathing or swallowing of the animal. 

In conclusion, our work demonstrated benefits in improving image quality on high-field animal EPI for different kinds of functional imaging like fMRI and DWI imaging. The PSF data acquisition and correction procedure works reliably and quickly and the additional acquisition time needed is worth the enhancement in geometric accuracy of the reconstructed images.

## List of Abbreviations

ADC: Apparent Diffusion Coefficient
BW: Bandwidth
DiCo: Distortion Correction
DTI: Diffusion Tensor Imaging
DW: Diffusion Weighting
DWI: Diffusion Weighted Imaging
EC: Eddy Currents
ECG: Electrocardiogram
EPI: Echo Planar Imaging
fMRI: functional MRI
FOV: Field of View
GE: Gradient Echo
MRI: Magnetic Resonance Imaging
PE: Phase Encoding
PSF: Point Spread Function
PSM: Pixel Shift Map
RF: Radio Frequency
rFOV: reduced FOV
SE: Spin Echo
TA: Acquisition Time
TE: Echo Time
TR: Repetition Time.
